# A survey of tuberculosis infection control practices at the NIH/NIAID/DAIDS-supported clinical trial sites in low and middle income countries

**DOI:** 10.1186/s12879-016-1579-y

**Published:** 2016-06-10

**Authors:** Catherine Godfrey, Gail Tauscher, Sally Hunsberger, Melissa Austin, Lesley Scott, Jeffrey T. Schouten, Anne F. Luetkemeyer, Constance Benson, Robert Coombs, Susan Swindells, HIV Network Coordinating (HANC) TB Diagnostic Laboratory Working Division of AIDS, National Institute of Allergy and Infectious Diseases, Debra Benator, Constance Benson, Robert Coombs, Peggy Coulter, Janice Darden, Anne Marie Demers, Joan Dragavon, Constance Ducar, Kathleen Eisenach, Carrie Frye, Morgan Gapara, Catherine Godfrey, Amy James Loftis, Vandana Kulkarni, Christopher Lane, Daniella Livnat, Annie Luetkemeyer, Peter Meewes, Beverly Metchock, Kurt Michael, Mark Nicol, Savita Pahwa, Anne Purfield, Jeffrey Schouten, Lesley Scott, Dave Shugarts, Donna Smith, Wendy Stevens, Susan Swindells, Gail Tauscher, Nicole Tobin, Frances Whalen, Sharon Williams, Carolyn Yanovich

**Affiliations:** Division of AIDS, National Institute of Allergy and Infectious Diseases, 5601 Fisher’s Lane Room 9E49, MSC 9830, 20892-9830 Bethesda, MD USA; Office of HIV/AIDS Network Coordination, Seattle, WA USA; Department of Molecular Medicine and Haematology, School of Pathology, Faculty of Health Sciences, University of the Witwatersrand Johannesburg South Africa, Johannesburg, South Africa; Fred Hutchinson Cancer Research Center, Seattle, WA USA; University of California San Francisco, San Francisco, CA USA; University of California San Diego, San Diego, CA USA; Departments of Laboratory Medicine and Medicine, University of Washington, Seattle, WA USA; Department of Medicine, University of Nebraska Medical Center, Omaha, NE USA

**Keywords:** Infection prevention, HIV, AIDS, Resource limited, IC, Tuberculosis, Tuberculosis prevention, Low and middle income countries

## Abstract

**Background:**

Health care associated transmission of *Mycobacterium tuberculosis* (TB) is well described. A previous survey of infection control (IC) practices at clinical research sites in low and middle income countries (LMIC) funded by the National Institute of Allergy and Infectious Diseases (NIAID) conducting HIV research identified issues with respiratory IC practices. A guideline for TB IC based on international recommendations was developed and promulgated. This paper reports on adherence to the guideline at sites conducting or planning to conduct TB studies with the intention of supporting improvement.

**Methods:**

A survey was developed that assessed IC activities in three domains: facility level measures, administrative control measures and environmental measures. An external site monitor visited each site in 2013–2014, to complete the audit. A central review committee evaluated the site-level survey and results were tabulated. Fisher’s exact test was performed to determine whether there were significant differences in practices at sites that had IC officers versus sites that did not have IC officers. Significance was assessed at *p*</=.05

**Results:**

Seven of thirty-three sites surveyed (22 %) had all the evaluated tuberculosis IC (TB IC) elements in place. Sixty-one percent of sites had an IC officer tasked with developing and maintaining TB IC standard operating procedures. Twenty-two (71 %) sites promptly identified and segregated individuals with TB symptoms. Thirty (93 %) sites had a separate waiting area for patients, and 26 (81 %) collected sputum within a specific well-ventilated area that was separate from the general waiting area. Sites with an IC officer were more likely to have standard operating procedures covering TB IC practices (*p* = 0.02) and monitor those policies (*p* = 0.02) and perform regular surveillance of healthcare workers (*p* = 0.02). The presence of an IC officer had a positive impact on performance in most of the TB IC domains surveyed including having adequate ventilation (*p* = 0.02) and a separate area for sputum collection (*p* = 0.02)

**Conclusions:**

Specific and targeted support of TB IC activities in the clinical research environment is needed and is likely to have a positive and sustained impact on preventing the transmission of TB to both health care workers and vulnerable HIV-infected research participants.

**Electronic supplementary material:**

The online version of this article (doi:10.1186/s12879-016-1579-y) contains supplementary material, which is available to authorized users.

## Background

Time and attention to infection control (IC) activities improves patient and staff infection rates [1, 2] and reduces costs associated with health care [3]. In resource limited settings, the contribution of health care associated infections to patient morbidity may be underestimated and significant [4]. Health care associated transmission of *Mycobacterium tuberculosis* (TB) is well described in both patients and health care workers and outbreaks of tuberculosis including extensively drug resistant TB (XDR TB) have been linked to the health care environment in several resource limited settings [5, 6]. Prevention of health care associated TB transmission, therefore, is an urgent IC need in resource-limited settings.

Recommendations regarding facilities level management activities, administrative, and environmental IC measures and personal protection practices [7–9] have been developed by several agencies including the World Health Organization (WHO) and the US Centers for Disease Control (CDC). In the United States, regulations require an “active program for the prevention, control, and investigation of infections and communicable diseases” [10], and wide implementation of a variety of IC practices in the last 20 years has resulted in a substantial decrease in health care associated transmission of TB [7]. In resource limited settings, there are no regulatory requirements, and uptake of guidance has been incomplete. Adherence to international guidelines has been evaluated in several settings and compliance with the full complement of recommendations is rare. In particular the presence of a written IC plan, staff and patient education, segregation of patients with symptoms and yearly evaluation of health care workers are uncommonly found [11–13]. Two recent studies in South Africa assessed the impact of health care worker education about IC principles and the results are worrisome [14, 15]. Although a higher level of training was associated with better knowledge of TB IC, this knowledge did not always translate into better TB IC (IC) practice [14]. Importantly, among health care workers (HCW) the rates of smear positive TB were higher than in the general population and the audit cycle (standard setting, evaluation of practice and outcomes and re-audit) had no impact on rates of incident TB in this group. A general review of IC practices including respiratory and hand hygiene, IC organizational structure and blood safety at NIAID funded clinical research sites was conducted in 2011 and 2012 [16]. Several important issues were identified at the sites in low and middle income countries, the most urgent of which was the need for a network policy for TB IC. A guideline for TB IC was developed based on existing international recommendations and promulgated early in 2012. External monitors were then sent to all sites to assess adherence to the guideline. This paper reports on uptake of these recommendations at clinical sites conducting or planning to conduct TB-related ACTG clinical trials with the objective of improving participant and staff safety.

## Methods

Thirty-three NIAID-funded clinical research sites located in LMIC in the AIDS Clinical Trials Group (ACTG) and the International Maternal, Pediatric, Adolescent AIDS Clinical Trials Network (IMPAACT) networks signaled their intention to participate in clinical trials involving TB. These sites were given the ACTG TB IC guideline, and a survey was developed to assess adherence to the ACTG guideline [Additional file 1]. This survey tool was piloted at five representative clinical trial sites and refined to improve the ease of its administration for both the monitor and the site staff as well as to address minor technical and scientific issues. The sites were then monitored by an objective external monitoring organization (PPD) to assess adherence to the guideline using the survey. The external monitoring organization visited each site and directly observed IC practice between February 2013 and December 2014 and documented adherence to the ACTG guideline using the survey tool. The results of each site survey were reviewed by an ACTG committee in real time, and feedback was given to each site in order to improve practice.

Notionally the survey was divided into three key components of TB IC practice: facility level measures, administrative control measures and environmental control measures (see Table 1). Facilities level activities that were evaluated included whether an air flow assessment for clinic areas had been performed, whether patients with symptoms and cough were identified and separated, and whether sputum collection was separate from the patient care areas and collected in an appropriate facility. Waiting areas were evaluated for crowding and ventilation. The presence of policies for annual TB surveillance of health care workers (HCW) and policies for HIV Post-Exposure Prophylaxis (PEP) and Isoniazid Preventive Therapy (IPT) were noted as well as quality management practices for regular assessment of adherence to written policies and standard operating procedures. External monitors assessed whether personal protective equipment (PPE) including N95 masks or equivalent were available and fit tested. Policies for hand hygiene and appropriate instruction were recorded as present or absent.

**Table 1:** Components of the site audit tool and response rates from 32 sites

Key components	Indicators	Details	Result
Facility level measures	Facility plan for implementation of TB infection control	Appointment of facility based IC officer	61.0 %
		Policies and procedures (SOPs) for rapid identification, and isolation of TB cases	61.3 %
	Optimized space	Waiting area ventilated and uncrowded	93.3 %
	Annual surveillance of health care workers (HCWs) is conducted	Any or all of: Symptom screen/TB skin test/Interferon-gamma Release Assay (IGRA)/Chest x-ray	61.3 %
	TB IC Policies monitored	1. Policies evaluated	80.6 %
		2. Documentation of frequency of training, training materials updated	
Administrative control measures	Identification and separation of patients with symptoms	1. Identification and separation of potentially infectious patients, including written procedures	71 %
		2. Use of separate waiting rooms or outdoor waiting areas	
	Cough hygiene Education	1. Cough hygiene education signs	80.6 %
		2. Resources for hand washing/disinfection	
		3. Masks for coughing patients and others with respiratory infection.	
	Hand hygiene Policy	1. Written policy or appropriate instructions for hand hygiene	67.7 %
		2. Instructions posted	
		3. Availability and use of non-touch bins throughout the high-risk areas	
	Separation of sputum collection	Separate collection area	80.6 %
		Well-ventilated area	
Environmental control measures	Ventilation system is optimized	Air flow assessment performed	54.8 %
	Ventilation and/or air cleaning	Windows open, use of other ventilation, air cleaning methods	80.6 %
	Personal Protective Equipment (PPE) (PPE, N95 masks or equivalent, fit testing for masks)	PPE available	96.8 %
		(N95 Masks), gloves and respirators	87.1 %
		Fit Testing: written fit testing procedures, records for fit testing	43.3 %

Two reviewers (CG and GT) summarized and tabulated the information, percentages of positive responses were recorded and a yes/no assessment to open ended questions was provided. The answers to the elements in the assessment tool provided a measure of adherence to the ACTG IC guideline. A Fisher’s exact test was performed to determine whether there were significant differences in practices at sites that had IC officers versus sites that did not have IC officers. Significance was assessed at *p*</=.05

No ethical review was required as this was not research involving human subjects.

## Results

Thirty one of thirty-three sites had complete responses to the IC survey. Seven sites (22 %) had all the surveyed elements already in place.

### Facilities level control

Sixty-one percent of sites had an IC officer tasked with developing and maintaining SOPs. Just over half the sites had SOPs in place for the major IC domains, and 81 % (25/31) of these performed and documented regular audits of their SOPs. Health care workers were required to have annual screening for tuberculosis at 61 % (19/31) of sites. Screening could consist of any of a symptom screen, a CXR or a tuberculin skin test.

### Administrative

Most sites had some TB IC administrative support. Seventy-one percent of sites promptly identified individuals with TB symptoms and segregated them.

### Environmental

Ninety-three percent of sites (28/30) had a separate waiting area for patients, and collected sputum in a specific area (81 %) that was separate from the general area and well ventilated as judged by the auditor. Sites were likely to promote cough hygiene by having signs posted, available disposable handkerchiefs, and disposable masks for patients (81 % of sites). There was adequate ventilation with measurement of airflow in the waiting areas, examination rooms and specimen collection areas at 81 % of sites. Personal protective equipment was commonly present (97 %), including N95 masks and respirators (87 %), but masks were not commonly fit tested (43 %–13/30 sites).

The sites with an IC officer in general performed significantly better in most but not all of the domains surveyed (see Table 2). Specifically, sites with an IC officer were more likely to have standard operating procedures for important IC practices (*p* = 0.02; 95 % confidence intervals 9.0 %, 71.5 %) and to monitor adherence to these practices (*p* = 0.02; 6.2–63.9 %) were more likely to perform surveillance of health care workers (*p* = 0.02; 8.8–72.5 %). These sites were also more likely to have adequate ventilation (*p* = 0.02; 5.9– 64.2 %) and separate areas for sputum collection (*p* = 0.02; 6.0– 64.3 %).

**Table 2:** Role of IC officer

	Total	No officer	Officer	Difference in proportion (95 % confidence) between officer and no officer	*P*-value
IC SOPs	19 (61.3 %)	4 (33.3 %)	15 (78.9 %)	45.6 % (9.0, 71.5)	0.022
Sufficient waiting area space	28 (93.3 %)	10 (83.3 %)	18 (100 %)	16.7 % (−4.1, 42.5)	0.152
Surveillance of HCW	19 (61.3 %)	4 (33.3 %)	15 (78.9 %)	45.6 % (8.8, 72.5)	0.022
TB IC policies monitored	25 (80.6 %)	7 (58.3 %)	18 (94.7 %)	36.4 % (6.2, 63.9)	0.022
ID, Separation of patients with symptoms	22 (71 %)	7 (58.3 %)	15 (78.9 %)	20.6 % (−12.8, 52.6)	0.253
Separate sputum collection	25 (80.6 %)	7 (58.3 %)	18 (94.7 %)	36.4 % (6.0, 64.3)	0.022
Ventilation	25 (80.6 %)	7 (58.3 %)	18 (94.7 %)	36.4 % (5.9, 64.2)	0.022
PPE available	30 (96.8 %)	11 (91.7 %)	19 (100 %)	8.3 % (−8.0, 33.2)	0.387
N95 or equivalent masks	27 (87.1 %)	10 (83.3 %)	17 (89.5 %)	6.1 % (−18.3, 35.2)	0.63
Fit testing for masks	13 (43.3 %)	3 (25 %)	10 (55.6 %)	30.6 % (−6.0, 59)	0.141

Number and percent of positive responses for each domain by whether a site had an IC officer present. The p-value is a comparison of the proportions between sites with IC officers and without IC officers

## Discussion

The results of our study provide an assessment tool for continuing improvement in the area of TB IC in the clinical research setting. The clinical research environment provides an opportunity to model and evaluate best practices for clinical care. Typically the patient volume is less than in the clinical care setting and there are rigorous standards for good clinical practice with which all members of the research team are required to be certified. Indeed, many have argued that the research environment should be held to the highest achievable standard of clinical care [17] which may differ from local standard of care. Nevertheless, we identified important gaps in TB IC knowledge and areas for improvement of IC practices. Most of the NIAID Division of AIDS (DAIDS) sponsored clinical sites had some basic TB elements for IC practices in place and a few sites had all the important elements in place. The most important finding was that when a trained IC officer was present, the facility was more likely to have SOPs in place for the major TB IC domains, to segregate sputum collection from the general study participant areas, and to provide annual surveillance of health care workers for TB infection. The TB IC survey was used to make site-specific recommendations in real time for improvement of TB infection prevention practices and capacity building. For example, standardized instructions were developed for assessing airflow and inexpensive equipment for its measurement were sourced. Having the elements in place did not imply that the practices were the best available, but they provided a framework in which improvement could occur. For example, having a policy of yearly screening of health care workers already in place, means that the specifics of the screening can then be evaluated as a next step.

The effect of specific IC activities is difficult to measure but evaluation of adherence to specific recommendations will be a helpful next step. Despite appropriate TB IC training, it still may be difficult to prevent health care associated transmission of TB. The administrative component of IC practice such as the presence of an IC practitioner, policies and procedures for handling patients and staff members with infections and documentation of those procedures, has been demonstrated to lead to improved outcomes in resource rich environments [18, 19]. A review of the effect of IC interventions in low and middle income countries concluded that administrative controls have the potential to reduce new TB infections in health care workers [6]. There also may be an incremental benefit to engineered solutions such as the use of mechanical ventilation and ultraviolet (UV) lights to limit TB infectiousness [6]. In resource-limited settings high clinical burden, shortages of the materiel required for universal precautions (eg PPE), minimal support from health care management for these activities and minimal or no regulatory requirements for compliance to IC procedures make adhering to IC recommendations difficult. High staff turnover provides an additional challenge. The effect of a specific single intervention, is therefore harder to prove and is less well studied

Health care workers may be an important link in the transmission cycle of TB in clinical research settings: the risk to clinic staff for TB acquisition may be considerable and staff with active TB may put other vulnerable patients at risk. The prevalence of TB infection and disease is high in health care workers compared to the general population [6, 20]. Latent TB infection is common and in a prospective study, HCW experienced incident latent TB infection almost five times more than a control population [20]. A recent meta-analysis found that in high TB prevalence countries, 81 % of active TB disease cases in health care workers was attributable to exposure in their work environment [21]. When active surveillance of health care workers is undertaken, undiagnosed pulmonary disease is not uncommon, and of those with active pulmonary TB multidrug resistant strains comprise an important component [22–24]. Rates of admission to hospital for TB were evaluated in Kwa Zulu-Natal and the estimated incidence of MDR TB hospitalization for HCW was more than five times that of the non-health care worker population and HCW represented a significant proportion of the XDR cases [25]. Universal screening for TB at the workplace, undifferentiated by HIV status is now recommended by the WHO [26] although few resource limited countries undertake regular screening [6, 27] of any type.

Evaluation of adherence to specific recommendations will be a helpful next step at the DAIDS funded clinical research sites. Documentation of adherence to SOPs and other surveillance activities will identify other gaps at a site level and continued surveillance of the aggregate performance will provide important information that will allow for the deployment of targeted resources. Biologically relevant surrogate markers for assessing the success of IC measures remain elusive in the outpatient environment, although a decrease in new cases of TB infection and disease in HCW has been proposed as a measurement of TB IC success [15, 27–31]. Quantification on a population level of TB burden using various methodologies including Xpert probe frequency is a novel technique that may provide additional information [32].

## Conclusions

In our study the presence of an IC practitioner was more likely to be associated with the availability of basic elements of IC practice, however this was less influential on overall TB specific IC practices than we expected. It may be that the role and job description of the IC officer needs to be standardized and ownership of the principles by the entire site staff may be required. Some experts have argued that the implementation of administrative level controls such as the identification and isolation of individuals with cough and the provision of preventive and care services for health care workers should be prioritized [6, 11], however the contribution of each component of an effective IC program has not been evaluated and it may be that the presence of managerial support of resources including commitment to the establishment, implementation and monitoring of IC policies is critical. Our study lends credence to this notion, and suggests that support of an individual specifically tasked with IC activities may improve the overall quality of care at and reduce nosocomial transmission of infections to HCW and trial participants. Regular TB surveillance of health care workers, site study personnel and research participants is clearly an important component of IC practice in resource limited settings and may provide an outcome measure of IC activities. Inculcating a best practices culture of infection prevention and continuous quality improvement will help ensure that all site personnel adhere to and benefit from basic IC principles. A research agenda for TB IC in areas of high prevalence is critically needed in order to prioritize efforts and to focus attention on this acute need.

## Abbreviations

ACTG, AIDS Clinical Trials Group; CDC, centers for disease control; CXR, chest radiograph; DAIDS, NIAID Division of AIDS; HCW, Health Care Workers; HIV, Human Immunodeficiency Virus; IC, infection control; IMPAACT, International Maternal Pediatric, Adolescent AIDS Clinical Trials Network; IPT, isoniazid prevention therapy; LMIC, low and middle income countries; NIAID, National Institute of Allergy and Infectious Diseases; PEP, post-exposure prophylaxis; PPE, personal protective equipment; SOP, standard operating procedure; TB, *Mycobacterium tuberculosis*; UV, ultraviolet; WHO, World Health Organization

## Additional file

Additional file 1: Clinical site tuberculosis (TB) infection control checklist. (PDF 405 kb)
