# Supplementary material for: A survey of tuberculosis infection control practices at the NIH/NIAID/DAIDS-supported clinical trial sites in low and middle income countries
Source: BMC Infect Dis. 2016 Jun 10;16:269. doi: 10.1186/s12879-016-1579-y (PMC4901412; doi:10.1186/s12879-016-1579-y)
Supplement: Additional file 1: — Clinical site tuberculosis (TB) infection control checklist. (PDF 405 kb) [file 12879_2016_1579_MOESM1_ESM.pdf]

CLINICAL SITE TUBERCULOSIS (TB) INFECTION CONTROL CHECKLIST

**Name, Role, and Title of Person Completing the Checklist**			
**Site Number:**		**Checklist Completion Date:**	
**Site Name:**		**City, State, Country:**	
**Program Officer:**		**Clinical Trial Unit (CTU) Name:**	
**Clinical Research Site (CRS) Leader:**		**CTU Principal Investigator (PI):**	

REFERENCE ONLY

CLINICAL SITE TUBERCULOSIS (TB) INFECTION CONTROL CHECKLIST

I. Facility Level Measures				
**A. Has a plan been developed for the facility to ensure proper implementation of TB infection control which includes:**				
	**Yes***	**No****	**Suggested Guidance to Monitors:**<br>**Things to look for (additional required details in parenthesis)**	**Comments**<br>*** If any of the items are in place, provide details in the comments section**<br>**** If no, explain the site's plan to put measures in place**
1. Appointment of a facility-based infection control officer to conduct regular assessments.			A written job description (name of person and designation at site)	
			Standard Operating Procedure (SOP) for the assessments (document name and version number/date)	
			A written plan to implement and monitor infection control (document name and version number/date)	
2. Policies and procedures to ensure proper implementation of the controls.			Written procedures for implementation of controls (document name(s) and version number(s)/date(s))	
3. A policy developed to support rapid identification, and isolation of TB cases in accordance with the ACTG TB Site Infection Control Guidelines.			Written procedures that meet the recommendations in the ACTG Site TB Infection Control Guidelines (document name(s) and version number(s)/date(s))	

CLINICAL SITE TUBERCULOSIS (TB) INFECTION CONTROL CHECKLIST

I. Facility Level Measures (Continued)				
B. Has space been optimized and evaluated for TB infection control which includes:				
	**Yes***	**No****	**Suggested Guidance to Monitors:** Things to look for (additional required details in parenthesis)	**Comments** * If any of the items are in place, provide details in the comments section ** If no, explain the site's plan to put measures in place
1. Current air flow assessment performed internally or by a professional.			Written records of such a technical assessment (name of person and designation at site , if internal or organization, if external professional, frequency of assessments, and date of last assessment)	
2. Sufficient space available in waiting area(s).			Describe space and render judgment as to whether it is sufficient or not. Consider crowding and whether people are wandering around	
3. Renovation of current space.			Written plans for renovation of current space (describe plans including estimated completion time)	
4. Planned construction for new space.			Written plans for construction of new space (describe plans including estimated completion time)	

REFERENCE COPY

CLINICAL SITE TUBERCULOSIS (TB) INFECTION CONTROL CHECKLIST

**I. Facility Level Measures (Continued)**				
**C. Is annual surveillance of health care workers (HCWs) conducted for TB infection and active disease to include:**				
	**Yes***	**No****	**Suggested Guidance to Monitors:** **Things to look for (additional required details in parenthesis)**	**Comments** *** If any of the items are in place, provide details in the comments section** **** If no, explain the site's plan to put measures in place**
1. Symptom screen			Example of a health assessment questionnaire or any other document used for this assessment (document name and version number/date)	
2. TB skin test or Interferon-gamma Release Assay (IGRA)			Example of a health assessment questionnaire or any other document used for this assessment (document name and version number/date)	
3. Chest x-ray (*not recommended annually for asymptomatic persons*)			Example of a health assessment questionnaire or any other document used for this assessment (document name and version number/date)	
4. Isoniazid Preventative Therapy			Written description of available isoniazid preventative therapy (document name and version number/date)	
			Communication or training materials for informing HCWs (if these exist, describe how they are distributed to HCWs)	
			Written procedures for informing HCWs and providing isoniazid preventative therapy (document name(s) and version number(s)/date(s))	

REFERENCE CO

CLINICAL SITE TUBERCULOSIS (TB) INFECTION CONTROL CHECKLIST

I. Facility Level Measures (Continued)				
D. Is a package of prevention and care interventions available for HCWs which includes:				
	**Yes***	**No****	**Suggested Guidance to Monitors:** Things to look for (additional required details in parenthesis)	**Comments** * If any of the items are in place, provide details in the comments section ** If no, explain the site's plan to put measures in place
1. Availability of HIV testing to HCWs			In-country guidelines for HIV testing of HCWs (if guidelines exist, document name and version number/date. If no guidelines, describe how HCWs generally access HIV testing)	
			Example(s) of standardized forms for collecting results of HIV testing of HCWs (document name and version number/date)	
			Summary of results among HCWs (document name and version number/date)	
2. HIV Prevention			Written description of available prevention methods for HIV (document name and version number/date)	
			Communication or training materials for informing HCWs (if these exist, describe how they are distributed to HCWs)	
			Written procedures for informing HCWs and providing HIV prevention (document name(s) and version number(s)/date(s))	
3. Antiretroviral Therapy			Written description of available ART (document name and version number/date)	

REFERENCE COPY

CLINICAL SITE TUBERCULOSIS (TB) INFECTION CONTROL CHECKLIST

I. Facility Level Measures (Continued)				
**D. Is a package of prevention and care interventions available for HCWs which includes:**				
	**Yes***	**No****	**Suggested Guidance to Monitors:** Things to look for (additional required details in parenthesis)	**Comments** * If any of the items are in place, provide details in the comments section ** If no, explain the site's plan to put measures in place
			Communication or training materials for informing HCWs (if these exist, describe how they are distributed to HCWs)	
			Written procedures for informing HCWs and providing ART (document name(s) and version number(s)/date(s))	

REFERENCE ONLY

CLINICAL SITE TUBERCULOSIS (TB) INFECTION CONTROL CHECKLIST

I. Facility Level Measures (Continued)				
E. Are TB infection control measures:				
	**Yes***	**No****	**Suggested Guidance to Monitors:**<br>**Things to look for (additional required details in parenthesis)**	**Comments**<br>*** If any of the items are in place, provide details in the comments section**<br>**** If no, explain the site's plan to put measures in place**
1. Monitored and/or evaluated by site personnel			Records of internal review or other monitoring/evaluation activity (name of person performing the review, designation at site, frequency of reviews and date of last review)	
2. Inclusive of ongoing staff training and education on infection control measures and practices?			Training materials and records of completion	
			Summary of the title/publisher of training program and frequency of training	
3. Inclusive of TB specific infection control education materials (***for example, posters and/or pamphlets***) predominantly displayed in the waiting rooms and at strategic points within the facility?			Posters/pamphlets/other materials in the waiting rooms or other places in the facility	

REFERENCE CO

CLINICAL SITE TUBERCULOSIS (TB) INFECTION CONTROL CHECKLIST

**II. Administrative Level Measures**				
**A. Do concerted efforts to optimize administrative control include:**				
	**Yes***	**No****	**Suggested Guidance to Monitors:**<br>**Things to look for (additional required details in parenthesis)**	**Comments**<br>*** If any of the items are in place, provide details in the comments section**<br>**** If no, explain the site's plan to put measures in place**
1. Prompt identification of individuals with TB symptoms?			Written procedures that include the prompt identification of individuals with TB symptoms (document name(s) and version number(s)/date(s))	
2. Separation of potentially infectious patients?			Written procedures that includes the separation of potentially infectious patients (document name(s) and version number(s)/date(s))	
			Existence of room(s) or outdoor area(s) where potentially infectious patients are asked to wait (describe space)	
3. Controlling the spread of pathogens **(*cough etiquette and respiratory hygiene*).**			Signs posted at entrances with instructions to patients with symptoms of respiratory infection to:<br>• Cover mouths/noses when coughing/sneezing<br>• Use and dispose of tissues<br>• Wash or disinfect hands after they've been in contact with respiratory secretions	
			Tissues and no-touch receptacles for disposal of tissues provided in or near waiting area(s)	
			Resources for washing/disinfecting hands in or near waiting area(s)	

CLINICAL SITE TUBERCULOSIS (TB) INFECTION CONTROL CHECKLIST

**II. Administrative Level Measures**				
**A. Do concerted efforts to optimize administrative control include:**				
	**Yes***	**No****	**Suggested Guidance to Monitors:** **Things to look for (additional required details in parenthesis)**	**Comments** *** If any of the items are in place, provide details in the comments section** **** If no, explain the site's plan to put measures in place**
			Masks available to coughing patients and other people with signs/symptoms of respiratory infection at entrance	
4. Minimizing the time patients spend in health care facilities?			Written policies for minimizing the time patients spend in the facility (document name(s) and version number(s)/date(s))	
			Records of efforts made to reduce the time patients spend in the healthcare facility (if efforts are documented, provide document name and version number/date, if not, describe briefly)	

REFERENCE ONLY

II. Administrative Level Measures (Continued)				
B. Is collection of sputum samples:				
	**Yes***	**No****	**Suggested Guidance to Monitors:** Things to look for (additional required details in parenthesis)	**Comments** * If any of the items are in place, provide details in the comments section ** If no, explain the site's plan to put measures in place
1. Separate from the general waiting area?			Existence of sputum collection area separate from the general waiting area (describe space)	
			Written procedures and/or map indicating sputum collection area separate from the general waiting area (document name(s) and version number(s)/date(s))	
2. Are special precautions taken if the patient has known or suspected drug-resistant TB?			Written procedures for identifying and managing patients with known or suspected drug-resistant TB (document name(s) and version number(s)/date(s))	
3. Performed in well-ventilated area, room, booth or outside in the open air?			Well-ventilated area, room, booth, or open air area where sputum is collected. Well-ventilated means with natural ventilation (open windows with cross breeze), exhaust systems (for example, extractor fan at window), mechanical ventilation, or open air area (describe space)	

REFERENCE COPY

CLINICAL SITE TUBERCULOSIS (TB) INFECTION CONTROL CHECKLIST

**III. Environmental Level Measures**				
**A. Has a plan been developed for the facility to ensure proper implementation of TB infection control which includes:**				
	**Yes***	**No****	**Suggested Guidance to Monitors:**<br>**Things to look for (additional required details in parenthesis)**	**Comments**<br>*** If any of the items are in place, provide details in the comments section**<br>**** If no, explain the site's plan to put measures in place**
1. The use of different types of ventilation systems such as natural, exhaust, or mechanical ventilation.			Natural ventilation with cross breeze (open windows or doors with cross breeze)/ Exhaust systems (for example, extractor fan at window)/<br>Mechanical ventilation/ Outdoor area<br>(indicate ventilation system(s) used and area [for example, natural ventilation in waiting area and extractor fan in examination and specimen collection rooms])	
2. Air cleaning methods such as ultraviolet germicidal irradiation or the use of in-room air cleaning devices such as a HEPA filter unit (if adequate ventilation cannot be achieved).			Upper room UV lights/ HEPA filter units/ other in-room air cleaning devices (indicate air cleaning method(s) used and area, for example, UV lights and HEPA filter unit in examination and specimen collection rooms)	

REFERENCE COPY

CLINICAL SITE TUBERCULOSIS (TB) INFECTION CONTROL CHECKLIST

III. Environmental Level Measures (Continued)				
B. Has space been optimized and evaluated for TB infection control which includes:				
	**Yes***	**No****	**Suggested Guidance to Monitors:** Things to look for (additional required details in parenthesis)	**Comments** * If any of the items are in place, provide details in the comments section ** If no, explain the site's plan to put measures in place
1. Ongoing procurement of necessary PPE for infection control.			Stocks of appropriate PPE, such as masks, respirators, gloves, lab coats, et cetera (list PPE in stock)	
			Ordering records for these items showing that they are ordered regularly (if records are not available at the site, indicate where they are kept and if copies can be made available for the site)	
2. Availability of respirators equivalent or greater than US-certified N95 or EU-certified FFP2 to all HCWs providing care to patients/research participants who may have TB.			Existence of such respirators readily available to HCWs (describe type of respirator, for example, non-powered respirators with N95 filters)	

REFERENCE CO

III. Environmental Level Measures (Continued)				
B. Has space been optimized and evaluated for TB infection control which includes:				
	**Yes***	**No****	**Suggested Guidance to Monitors:** Things to look for (additional required details in parenthesis)	**Comments** * If any of the items are in place, provide details in the comments section ** If no, explain the site's plan to put measures in place
3. Disposable respirators are properly fit tested for HCWs in situations where there is an increased risk of TB transmission.			Written procedures for such fit testing (document name(s) and version number(s)/date(s))	
			Training materials and/or records for such fit testing (If training materials exist, describe how they are distributed to HCWs. If records are not available at the site, indicate where they are kept and if copies can be made available for the site)	
4. An administrative policy is in place for use of respirators in high risk situations.			Written policy for use of respirators in high-risk situations (document name and version number/date)	
			Training materials and/or records for use of respirators (If training materials exist, describe how they are distributed to HCWs. If records are not available at the site, indicate where they are kept and if copies can be made available for the site)	
5. A proper hand washing policy is available and widely implemented throughout the institution			Written policy for proper hand washing (document name and version number/date)	
			Instructions for proper hand washing posted at hand washing stations	

CLINICAL SITE TUBERCULOSIS (TB) INFECTION CONTROL CHECKLIST

**III. Environmental Level Measures (Continued)**				
**B. Has space been optimized and evaluated for TB infection control which includes:**				
	**Yes***	**No****	**Suggested Guidance to Monitors:** Things to look for (additional required details in parenthesis)	**Comments** * If any of the items are in place, provide details in the comments section ** If no, explain the site's plan to put measures in place
6. Availability and use of non-touch bins throughout the high-risk areas.			Existence of non-touch bins and absence of touch bins throughout high-risk areas (list the areas)	

REFERENCE COPY
